# Epiphytic Orchid Diversity along an Altitudinal Gradient in Central Nepal

**DOI:** 10.3390/plants10071381

**Published:** 2021-07-06

**Authors:** Binu Timsina, Pavel Kindlmann, Sajan Subedi, Subhash Khatri, Maan B. Rokaya

**Affiliations:** 1 Global Change Research Centre, Department of Biodiversity Research, Czech Academy of Sciences, Bělidla 4a, 60300 Brno, Czech Republic; pavel.kindlmann@centrum.cz; 2 Institute for Environmental Studies, Faculty of Science, Charles University, Benátská 2, 12801 Prague, Czech Republic; 3 Botanics Nepal, Annapurna Marg-32, Kathmandu 44604, Nepal; sferox@gmail.com; 4 National Herbarium and Plant Laboratories, Post Box No. 3708, Godawari-5, Lalitpur 44709, Nepal; subhger99@yahoo.com; 5 Institute of Botany, Czech Academy of Sciences, Zámek 1, 25243 Průhonice, Czech Republic

**Keywords:** ecology, environmental factors, host, Orchidaceae, diversity pattern

## Abstract

Epiphytic orchids are common in subtropical forests, but little is known about the factors that determine their diversity. We surveyed two sites (north-facing Phulchowki and south-facing Shivapuri hills), in the sub-tropical forest in the Kathmandu valley, central Nepal. Along five transects per site, spanning an altitudinal gradient of 1525–2606 m a.s.l., we recorded all epiphytic orchids and the host species on which they were growing. The data were analyzed using a generalized linear model (GLM) and redundancy analysis (RDA). Species richness significantly decreased with increasing altitude and was higher in larger hosts and in places with high temperature. Species composition was affected by altitude, distance from the forest edge, host type, and precipitation. This study indicates that the most important factors affecting epiphytic orchid diversity was altitude, even if other factors were associated with patterns in composition. The low-altitude habitats with high species diversity are the best places for epiphytic orchids in this region. The altitudinal species richness and patterns in composition revealed by this study provide a baseline for further studies on epiphytic orchids.

## 1. Introduction

Diversity of plants and patterns in composition are commonly studied in order to understand the mechanisms underlying community assembly [1,2] and the underlying mechanisms determining the patterns [3,4,5]. Numerous factors directly or indirectly determine diversity and composition [6,7,8]; these may be, for example: (1) climatic variables, such as humidity, precipitation, temperature, etc. [5,9,10,11]; (2) edaphic variables, such as substrate, soil types, soil nutrients, etc. [1,12]; (3) geographical variables, such as altitude, latitude, longitude, etc.; (4) topographical variables, such as aspect, distance from forest edge, heat load index, ruggedness, slope, etc. Additional factors that determine the diversity of epiphytic species of plants are host characteristics such as bark pH, bark rugosity, stem diameter, type of host (deciduous/evergreen), host habit (shrub/trees), host age, host height, canopy cover, bark water holding capacity, etc. [13,14,15,16].

Many plant species, including orchids, are widely threatened because of habitat degradation due to agriculture, forestry, construction and mining, illegal trade, unsustainable utilization, and climate change [17,18,19]. In addition to this, many orchids are either threatened or rare because of their existence in small population sizes, limited distributions, and species-specific symbioses with pollinators and mycorrhizal fungi [20,21,22]. The latest update of the International Union for Conservation of Nature (IUCN) Global Red List released in July 2020 [19] includes assessments for 1641 orchid species (about 6% of orchids), of which five are categorized as extinct and 747 as threatened to various degrees: 197 Critically Endangered (CR), 355 Endangered (EN), 195 Vulnerable (VU). A further 87 species are categorized as Near Threatened (NT), 575 as Least Concern (LC), and 227 species are categorized as Data Deficient (DD), for which threat status is unknown. The abovementioned report shows that there is still a big gap in understanding different orchid species in terms of their distribution, ecology, and factors affecting their life cycle [23]. It has been emphasized that a good knowledge and understanding of species distribution patterns of different species is the basis for species conservation [24]. Out of different forms of orchids (saprophytes, lithophytes, epiphytes, and terrestrials), epiphytic orchids, which are the most abundant ones, can be studied in terms of their spatial distribution [25], the relationship of epiphytic orchids to host tree types and their characteristics [13,15], and environmental factors affecting them [26] to prioritize different species and develop effective strategies for their conservation [18,25].

The family Orchidaceae is one of the largest in the world and includes many epiphytic species, which occur in most places in the world [27,28], including Nepal—a country known for its rich biodiversity [29,30]—where Orchidaceae represent the second largest family. Epiphytic orchids grow mainly on different host types, such as trees, shrubs, evergreens, deciduous plants, etc. [15,31]. They are excellent biological indicators of ecological changes in the ecosystem [32]; thus, the pattern in species richness and composition of epiphytic orchids are well studied, particularly in the Himalayas [13,15,25]. In addition to this, epiphytic orchids are important in horticulture because of their beautiful flowers [33], medicinal properties [34,35], and also as vital components of biodiversity [36,37]. Studies have shown that the numbers of species generally decline with increase in altitude [25], which is associated with declines in temperature, precipitation, and soil fertility, along with a reduction in the growth season and low energy input [11,13,15,38,39]. However, a fine scale survey of epiphytic orchids along an altitudinal gradient including all other possible factors will enable us to acquire a deeper knowledge that can be used in the management and conservation of epiphytic orchids [18,40].

In Nepal, studies on epiphytic orchids focus mainly on (i) taxonomy [30,41,42,43], (ii) medicinal values [44,45], (iii) micropropagation [46], (iv) distribution at large spatial scales [25], (v) associations between diversity and distribution and host characteristics at different localities [13,15], (vi) diversity under different forest management regimes [13] or along land-use gradients [47], and (vii) conservation issues [48]. There has, however, not been a fine scale field survey of epiphytic orchids along an altitudinal gradient in Nepal. However, many other studies have shown that epiphytic vascular plants have high species richness in low altitudes, mainly below 1500 m a.s.l. [49,50]. In addition to this, though there are several studies related to different plant groups along an altitudinal gradient in Nepal [25,50,51,52,53,54,55,56,57] and in India [23,58,59,60,61], epiphytic vascular plants along an altitudinal gradient are widely neglected.

In this paper, we aim to survey epiphytic orchids along an altitudinal gradient on the north-facing Phulchowki and south-facing Shivapuri hills in the Kathmandu valley, Nepal, and explore types of environmental factors that affect species richness and species composition of surveyed epiphytic orchids. Specifically, our main aims are:

(a) To find out the species richness pattern of epiphytic orchids along an altitudinal gradient;
(b) To find out the species community composition (beta-diversity) of epiphytic orchids along an altitudinal gradient;
(c) To evaluate environmental variables that affect the observed altitudinal pattern and the importance of environmental variables in accounting for the species richness and composition of epiphytic orchids in the system studied.

## 2. Material and Methods

### 2.1. Study Area

**Figure 1:**
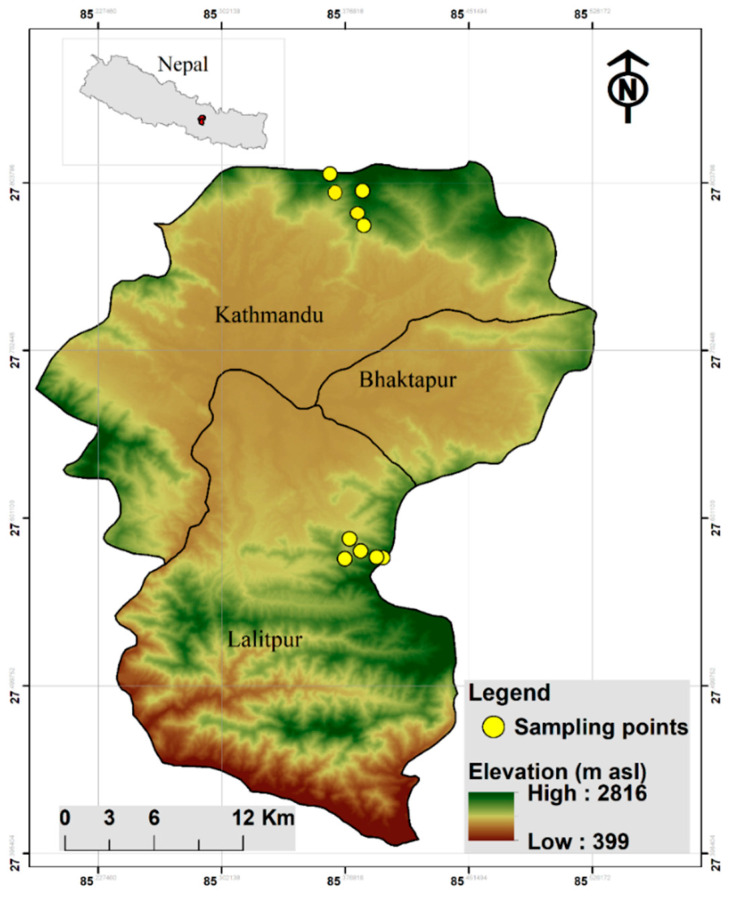
Map showing the locations of the areas in the Kathmandu valley, central Nepal, that were studied.

This study was conducted in subtropical forests in the Kathmandu valley, Nepal. Kathmandu valley is rich in terms of biodiversity [62,63]. However, there is a large human population in and around the valley so there is very little undisturbed forest there [64,65]. Prior to choosing the study sites, we surveyed different forests from low to high altitudes. Several places around Kathmandu were very poor in epiphytic species diversity because they were widely destroyed prior to the 1980s for collecting timber and firewood [66]. Thus, after visiting different places and consulting the vegetation map of Kathmandu valley, we finally decided to survey two sites, Shivapuri Nagarjun and Phulchowki, as they were rich in forest diversity, mainly with old trees, and we expected to find a high number of epiphytic orchids there. Anticipating differences in epiphytic orchids in different slope aspects along comparable altitudinal gradient, we selected two study sites in the subtropical forest in Kathmandu valley in central Nepal. Thus, the main criteria for selection of sites were that they were comparable in altitude, but different in their slope aspects. Specifically, the selected sites were: (i) the north-facing slope on Phulchowki hill, and (ii) the south-facing slope on Shivapuri hill, inside the Shivapuri Nagarjun National Park (Figure 1). This approach of comparing plant species was previously used between two different slope aspects in the same valley in high altitude in Nepal [67]. Daily temperature here is 17–27 °C in summer and 2–18 °C in winter, and average annual rainfall is 1400 mm [68]. Dominant trees in both areas are *Schima wallichii* (DC.) Korth, *Quercus lanata* Sm., *Castanopsis indica* (Roxb. ex Lindl.) A. DC., and *Rhododendron arboreum* (Sm.). Other associated trees are *Quercus semecarpifolia* Sm., *Myrica esculenta* Buch.-Ham. ex D. Don., *Lyonia ovalifolia* (Wall.) Drude and *Pinus roxburghii* Sarg. Average canopy cover in this forest is approximately 60% [47].

### 2.2. Data Collection

From August to mid-December 2020, at each of the two sites studied, along five line transects at each site, equidistantly spaced (approximately around 300 m) along the altitudinal gradient (1525 to 2412 m a.s.l. on Phulchowki and 1868 to 2606 m a.s.l. on Shivapuri hill) to cover the maximum possible range in altitude from the lowest undisturbed part of the study area to the top of the study sites. In each line transect, we sampled approximately 40 host species, mainly trees, but also some shrubs with epiphytic orchids. We used the horizontal line transect without any width in our survey because this approach was previously used in the study of epiphytic orchids in Nepal [15]. All the epiphytic orchids and their respective host trees were determined and noted down.

During the monitoring, we carefully observed the host from the ground to make sure that no orchids were overlooked. Most of the orchids were identified in the field using available field guides [29,37,44,69,70]. Plants that we were unable to identify on the spot were photographed and collected as herbarium specimens. They were later identified in the National Herbarium and Plant Laboratories (KATH) and also with the help of orchid taxonomist, B.B. Raskoti. Similarly, all hosts were identified in the field with the help of published field guides [71,72,73,74]; hosts that were not identified were later identified by visiting KATH. The nomenclature of epiphytic orchids is based on *An annotated checklist of the orchids of Nepal* [30,75,76] and that of the hosts on *Annotated Checklist of the Flowering Plants of Nepal* [75,76,77]. However, these two checklists are based on different international checklists, such as World Checklist of Selected Plant Families [78], The Plant List (TPL) [75] and Catalogue of Life [76]. Thus, names in the abovementioned checklists are updated.

For each host sampled, we recorded trunk diameter at breast height (DBH, i.e., diameter at 1.30 m), host height, nature of host (deciduous/evergreen), bark roughness in three categories (smooth, medium smooth, and rough) based on previous studies [15,47], canopy cover, distance from the nearest open road in the forest (i.e., forest edge), and altitude of the site. Mean annual temperature and annual precipitations were downloaded from WorldClim [79].

The environmental variables (used as explanatory variables in all analyses) and how they were collected are summarized in Table 1.

### 2.3. Data Analysis

#### 2.3.1. Terms Used in the Analysis

For the analysis, we calculated: (1) orchid species richness (total number of epiphytic orchid species per host); (2) orchid abundance (total number of individuals of each epiphytic orchid species per host); and (3) orchid species composition (the spatial variation of epiphytic orchid species along altitudinal gradient, beta-diversity).

#### 2.3.2. Effect of Geographical Distance on Orchid Diversity

In this study, to analyze the effect of geographical distance (i.e., site coordinates) on orchid beta-diversity, we used generalized dissimilarity modeling (gdm) [80]. It is a nonlinear statistical regression analysis technique, useful to decipher patterns of beta-diversity and their possible relationships with explanatory variables (e.g., geographical distance). For modeling, we used gdm() syntax of the gdm package (Version 1.4.2.2, [81]) in R 4.0.0 [82].

#### 2.3.3. Species Richness, Abundance, and Composition

To find out correlations among pairs of predictors, we calculated the variance-inflation factors in the model by using vif function in the car library [83]. Locality and host species were found to be problematic (their scores were more than 10), and therefore they were dropped from the regression model.

To find the best subset of predictors in a regression model, we used the regsubsets functions in the leaps package [84]. The quality of the adjustment of the models was measured by the adjusted coefficient of determination (R^2^) and Mallow’s Cp [85]. After choosing the best subset of predictors, determinants of the abundances and species richness of epiphytic orchids were tested using generalized linear models (GLMs). We tested the effects of different variables (altitude, annual precipitation, annual mean temperature, distance from forest edge, host type, bark type, and diameter at breast height) on species richness and species abundance. The analyses were carried out using R 4.0.0 [82].

As gradient length was short (2.18), we used redundancy analysis (RDA). It is a method to extract and summarize the variation in response variables known as species composition that can be explained by different explanatory variables such as altitude, annual precipitation, annual mean temperature, distance from forest edge, host type, bark type, and diameter at breast height. RDA is a direct gradient analysis technique, which summarizes linear relationships between components of response variables that are "redundant" with (i.e., "explained" by) a set of explanatory variables [86]. The significance of explanatory variables was tested using the Monte Carlo permutation test (n = 4999). All tests were carried out using Canoco 5.04 [87]. In the final graph, only the most frequent epiphytic orchids and significant explanatory environmental variables are plotted.

## 3. Results

We recorded 44 species of epiphytic orchids (Table S1) from 1419 orchid individuals recorded growing on 38 species of hosts belonging to 21 families (Table S2). Epiphytic orchids were recorded growing on 200 host individuals on the Phulchowki hill and on 169 host individuals on the Shivapuri hill. The lower number of hosts on Shivapuri is due to the absence of epiphytic orchids at high altitudes. The most frequently occurring orchids were *Dendrobium longicornu* (12% out of all orchids), *Pholidota imbricata* (8%), *Pholidota articulata* (7%), *Eria spicata* (6%), *Vanda cristata* (6%), *Bulbophyllum leopardinum* (4%), and *Coelogyne corymbosa* (4%). Nearly all hosts were trees, only *Berberis asiatica* and *Wikstroemia canescens* were shrubs. Twenty-nine species of hosts were recorded on Phulchowki and 27 on Shivapuri. The most important host species that occurred throughout the altitudinal gradient were *Quercus glauca* and *Quercus semecarpifolia*.

GDM analysis showed that the ecological dissimilarity (Bray–Curtis distance) based on epiphytic orchid occurrence and abundance increases with geographic distance and the most rapid change in ecological dissimilarity is near the higher end of geographical distance (Figure S1).

**Figure 2:**
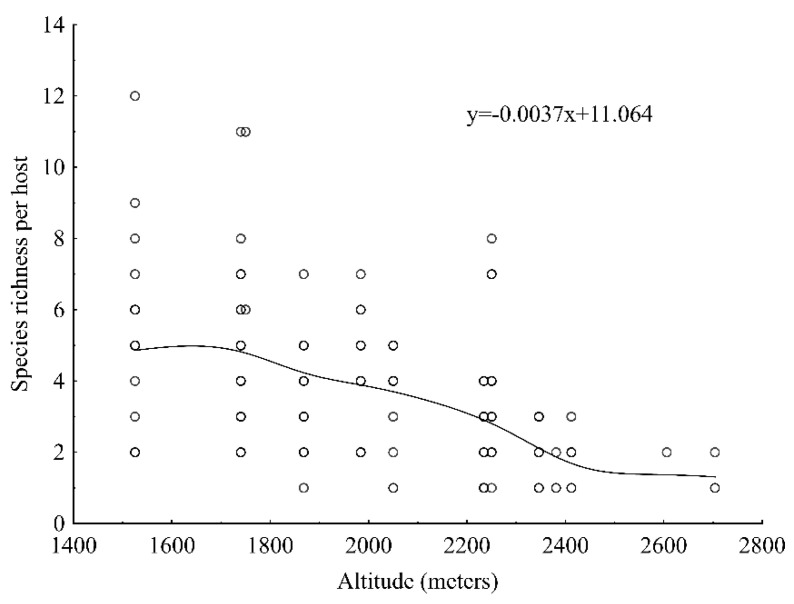
Relationship between orchid species richness per host and altitude.

Only orchid species richness decreased with increase in altitude (*p* < 0.001, R^2^ = 0.246) (Figure 2). Orchid species richness was higher on hosts with a large DBH (*p* < 0.01, R^2^ = 0.038) and species abundance was higher in areas with high temperature (*p* < 0.05, R^2^ = 0.044). However, the variation explained by DBH and temperature was very low.

**Figure 3:**
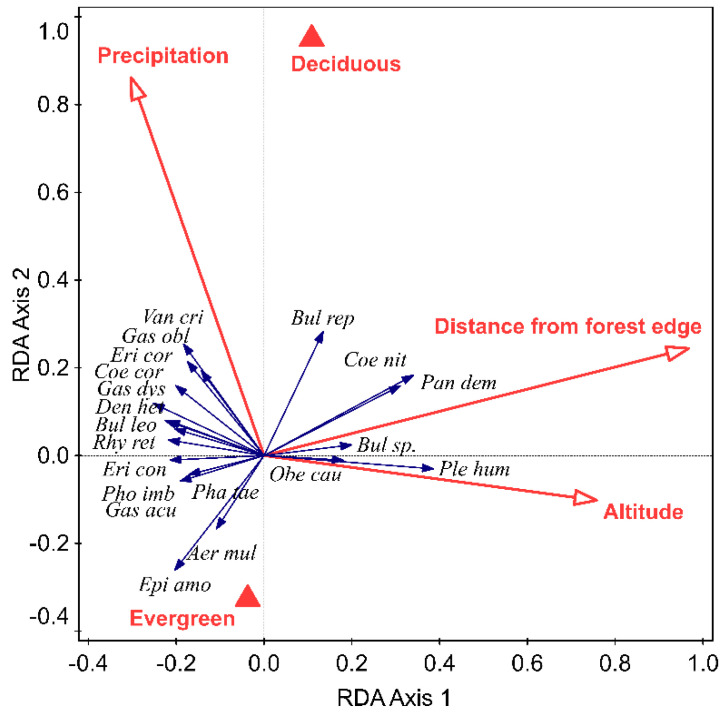
Relationship between different species of orchids and significant environmental variables (indicated by red arrows and red triangles). The 1st redundancy analysis (RDA) axis explained 4.24% and the 2nd 3.11% of the total variation in the data. Names in italics are species and in bold are environmental variables. See Tables S1 and S2 for full names of hosts and orchids.

The orchid species composition differed significantly between host types: evergreen and deciduous (*p* = 0.02). Most orchids, such as *Epigeneium amplum*, *Aerides multiflora, Phalaenopsis taenialis, Gastrochilus acutifolius, Pholidota imbricata,* and *Eria coronaria* were associated with evergreen hosts and few species, such as *Dendrobium longicornu* and *Bulbophyllum* sp., were associated with deciduous hosts. The orchid species composition was also significantly variable with altitude (*p* = 0.002), distance from the forest edge (*p* = 0.002), and annual precipitation (*p* = 0.004) (Figure 3).

Numbers of species of orchids per host decreased with increase in altitude and distance from the edge of the forest. The exception was *Pleione humilis*, *Coelogyne nitida*, *Panisea demissa,* and *Bulbophyllum* sp., which was frequently found far from the edge of the forest (i.e., deep in the forest) or at high altitudes (Figure 3). The few orchid species that were found in places with high precipitation were *Vanda cristata*, *Gastrochilus obliquus*, *Eria coronaria*, *Coelogyne corymbosa,* and *Gastrochilus dasypogon* (Figure 3).

## 4. Discussion

Recording of 44 species of epiphytic orchids in our study was much higher than in the previous study [13], and some of the epiphytic orchids such as *Agrostophyllum callosum, Aerides multiflora*, *Aerides odorata*, *Bulbophyllum purpureofuscum* were not even mentioned in local floras [71,88,89,90,91]. The present study indicated that fine scale sampling across a valley may result in the discovery of new species, e.g., the angiosperms (not orchids): *Hoya polyneura* [92] and *Thunbergia kasajuana* [93], which were recently reported in the vicinity of the Kathmandu valley. Not all hosts were trees, the most frequently occurring shrubs (*Berberis asiatica* and *Wikstroemia canescens*) in the forest undergrowth hosted a few species of orchids, such as *Eria spicata*, *Oberonia caulescens*, *Pholidota imbricata,* and *Pleione humilis*. This finding was similar to the previous study [15] and the reason for including shrubs as hosts of epiphytes is because once the shrubs are old enough, they have larger branches that are favorable for epiphytes to grow.

GDM analysis in our data set showed there were more dissimilar epiphytic orchid species at higher altitudes than at the lower altitudes, and the reason for having similar orchid species at lower altitudes is due to the presence of abundant trees and existence of favorable micro-climatic conditions than at higher altitudes [13]. In addition to this, similar radiation and water content are directly/indirectly associated with similar communities of plants at similar localities [13,15,94].

There was a significant decrease in orchid species richness with increasing altitude. However, the variation explained by altitude was relatively low, meaning that it is not the main factor responsible for the resultant pattern. Thus, in addition to altitude, there are other environmental factors that shape the patterns of diversity of epiphytic species. Decreasing of species numbers with increasing altitude was similar to that reported in other studies [95,96], but differed from the hump-shaped distribution reported by Acharya et al. [25] and in mid-altitude areas in Sabah, the northern part of Borneo [97]. The linear decrease recorded in this study is because the altitudinal range (1500–2700 m a.s.l.) studied was lower than that studied by Acharya et al. [25]: 100–5200 m a.s.l. and in Himachal Pradesh, where the majority of species were distributed between 1800–2800 m a.s.l. [60] or 2501–2800 m a.s.l. [61] or 1801–2800 m a.s.l. [59]. The observed differences in distribution patterns are because of differences in altitude coverage and differences in topography and climate of our and their study areas.

The variation in orchid species composition was similar to that reported in other studies from Nepal [13], Reunion Island [95] and Hainan Island, south China [1]. The variation in orchid species composition is brought about by differences in host types [13], topography, climate, and differences in the species of orchids recorded in different study areas [1,95]. In addition, the decrease in species diversity with increase in altitude is due to an increase in environmental harshness at high altitudes resulting from lower temperature [38], decreased soil fertility [98], and an increase in steep, rugged topography with little topsoil at high altitudes [99].

As mentioned earlier, large hosts (with big DBHs) have more area suitable for growth of epiphytic orchids [100] because they are more diverse in terms of having micro-microhabitats in their canopy [14,101,102], and they tend to support more species and/or individual epiphytic orchids because of having more space for growth.

Finding abundant species in places with high temperatures in our study is already a well-established fact [103], as warm areas support more species in low altitudes than in high altitudes [104] because the places with high temperatures are mostly favorable for species diversity.

The composition was affected by the distance from the forest edge. The fact that there were fewer species deep in the forest compared to the forest edge was similar to that reported in a semi-deciduous seasonal forest in the southeast of Brazil [105] and epiphytes in southern Brazil [106,107]. The reason for the effect of distance from the forest edges is because as one moves deeper into a forest, the forest gets thicker and less light penetrates below the canopy than at the edges of forest where the forest is not so dense, and more epiphytes growing at the edges of forests are diverse than in the deep thick forest [14,32,105,108,109].

## 5. Conclusions

The results of our study indicated that altitude is the main factor responsible for determining epiphytic orchids along an altitudinal gradient in the Kathmandu valley, central Nepal. In addition to this, the size of the host species and temperature slightly influence orchid diversity. Composition was affected by distance from the edge of the forest, annual precipitation, and deciduous and evergreen host types. It is clear that epiphytic orchids are an important component of local epiphytic species diversity; yet more work is needed to understand their ecology in order to maintain this biodiversity in the future. Our results indicate that the low-altitude areas with high epiphytic species and their host species also should be conserved and protected for future generations.

## Figures and Tables

**Table 1:** List of the explanatory environmental variables recorded at the sites studied.

Environmental Variable	Type of Data (Units)	Description
**Temperature**	Continuous (degrees centigrade)	Measure of hotness or coldness measured in Celsius and derived from WorldClim.
**Precipitation**	Continuous (mm)	Water released from clouds in different forms: as rain, freezing rain, sleet, snow, or hail. It was derived from WorldClim.
**Altitude**	Continuous (meters above sea level)	Height above sea level (m) measured using an altimeter.
**Distance from forest edge**	Continuous (meters)	Distance in meters measured using a measuring tape.
**Canopy cover**	Continuous (percentage)	Percentage cover of all host species using a densiometer.
**Nature of host (deciduous/evergreen)**	Categorical	Evergreen or deciduous.
**DBH**	Continuous (centimeters)	Host tree diameter at breast height taken at a height of 1.3 m using a measuring tape.
**Bark rugosity**	Ordinal [1,2,3]	Rugosity of host tree bark was determined visually. There are three classes as 1: Smooth, 2: Medium, 3: Rough.
**Host species**	Categorical (species type)	Identity of the host species with Latin names.

## Data Availability

The data presented in this study are available in the form of Supplementary Materials (Tables S1 and S3 and Figure S1). Detailed data are available upon request from the corresponding author.

## Supplementary Materials

The following are available online at https://www.mdpi.com/article/10.3390/plants10071381/s1, Table S1: List of epiphytic orchid species recorded in this study, Table S2: List of host species recorded in this study, Figure S1: Generalized dissimilarity model-fitted I-spline (partial regression fit) of geographical distance and ecological distance.
