# Supplementary material for: Epiphytic Orchid Diversity along an Altitudinal Gradient in Central Nepal
Source: Plants (Basel). 2021 Jul 6;10(7):1381. doi: 10.3390/plants10071381 (PMC8309340; doi:10.3390/plants10071381)
Supplement: Supplementary file 1 [file plants-10-01381-s001.zip › plants-1191543-supplementary.pdf]

Supplementary Materials

Table S1. List of epiphytic orchid species recorded in this study.

Orchid species	Abbreviations
Agrostophyllum callosum Rchb.f.	Agr cal
Aerides multiflora Roxb.	Aer mul
Aerides odorata Lour.	Aer odo
Bulbophyllum affine Lindl.	Bul aff
Bulbophyllum careyanum (Hook.) Spreng.	Bul care
Bulbophyllum cariniflorum Rchb.	Bul cari
Bulbophyllum leopardinum (Wall.) Lindl. ex Wall.	Bul leo
Bulbophyllum purpureofuscum J.J.Verm.	Bul pur
Bulbophyllum reptans (Lindl.) Lindl. ex Wall.	Bul rep
Bulbophyllum sp.	Bul sp.
Coelogyne corymbosa Lindl.	Coe cor
Coelogyne cristata Lindl.	Coe cri
Coelogyne flaccida Lindl.	Coe fla
Coelogyne fuscescens Lindl.	Coe fus
Coelogyne nitida (Wall ex. D. Don) Lindl.	Coe nit
Coelogyne ovalis Lindl.	Coe ova
Coelogyne prolifera Lindl.	Coe pro
Cymbidium elegans Lindl.	Cym ele
Cymbidium iridioides D. Don	Cym iri
Epigeneium amplum (Lindl.) Summerh.	Den amp
Dendrobium amoenum Wall. ex Lindl.	Den amo
Dendrobium denudans D. Don	Den den
Dendrobium eriiflorum Griff.	Den eri
Dendrobium heterocarpum Wall. ex Lindl.	Den het
Dendrobium longicornu Lindl.	Den lon
Dendrobium peguanum Lindl.	Den peg
Eria spicata (D. Don) Hand.-Mazz.	Eri con
Eria coronaria (Lindl.) Rchb. f.	Eri cor
Gastrochilus acutifolius (Lindl.) Kuntze	Gas acu
Gastrochilus calceolaris (Buch.-Ham. ex Sm.) D. Don	Gas cal
Gastrochilus dasypogon (Sm.) Kuntze	Gas dys
Gastrochilus obliquus (Lindl.) Kuntze.	Gas obl
Sunipia bicolor Lindl.	Sun bic
Sunipia cirrhata (Lindl.) P. F. Hunt	Sun cir
Oberonia caulescens Lindl.	Obe cau
Otochilus fuscus Lindl.	Oct fus
Panisea demissa (D. Don) Pfitzer	Pan dem
Pleione humilis (Sm.) D. Don	Ple hum
Pleione praecox (Lindl.) D. Don	Ple par
Phalaenopsis taenialis (Lindl.) Christenson & Pradhan	Pha tae

Pholidota articulata Lindl.	Pho art
Pholidota imbricata Hook.	Pho imb
Rhynchostylis retusa (L.) Blume	Rhy ret
Vanda cristata Wall. ex Lindl.	Van cri

Table S2 List of host species recorded in this study.

**Trees**	**Abbreviations**
Acanthopanax sp.	Aca sp.
Albizia chinensis (Osbeck) Merr.	Alb chi
Alnus nepalensis D. Don	Aln nep
Berberis asiatica Roxb. ex DC.	Ber asi
Betula alnoides Buch.-Ham. ex D. Don	Bet aln
Betula alnoides Buch.-Ham. ex D. Don	Bet aln
Castanopsis hystrix Miq.	Cas hys
Castanopsis indica (Roxb.) Miq.	Cas ind
Castanopsis tribuloides (Sm.) A. DC.	Cas tri
Choerospondias axillaris (Roxb.) B. L. Burtt & A. W. Hill	Cho axi
Cornus capitata Wall.	Cor cap
Eriobotrya dubia (Lindl.) Decne.	Eri dub
Eriobotrya japonica (Thunb.) Lindl.	Eri jap
Eurya acuminata DC.	Eur acu
Jasminum humile L.	Jas hum
Ligustrum indicum (Lour.) Merr.	Lig ind
Ligustrum sp.	Lig sp.
Lindera nacusua (D. Don) Merr.	Lin nac
Lindera pulcherrima (Nees) Benth. ex Hook. f.	Lin pul
Lithocarpus elegans (Blume) Hatus. ex Soepadmo	Lit ele
Lyonia ovalifolia (Wall.) Drude	Lyo ova
Meliosma dilleniifolia (Wall. ex Wight & Arn.) Walp.	Mel dil
Michelia sp.	Mic sp.
Myrica esculenta Buch.-Ham. ex D. Don	Myr esc
Pinus roxburghii Sarg.	Pin rox
Pinus wallichiana A. B. Jacks.	Pin wal
Prunus cerasoides D. Don	Pru cer
Pyrus pashia Buch.-Ham. ex D. Don	Pyr pas
Quercus glauca Thunb.	Que gla
Quercus lamellosa Sm.	Que lam
Quercus lanata Sm.	Que lan
Quercus semecarpifolia Sm.	Que sem
Rhododendron arboreum Sm.	Rho arb
Saurauia napaulensis DC.	Sau nap
Schima wallichii (DC.) Korth.	Sch wal
Symplocos theifolia D. Don	Sym the
Viburnum mullaha Buch.-Ham. ex D. Don	Vib mul
Wikstroemia canescens Meisn.	Wik can
Zizyphus incurva Roxb.	Ziz inc

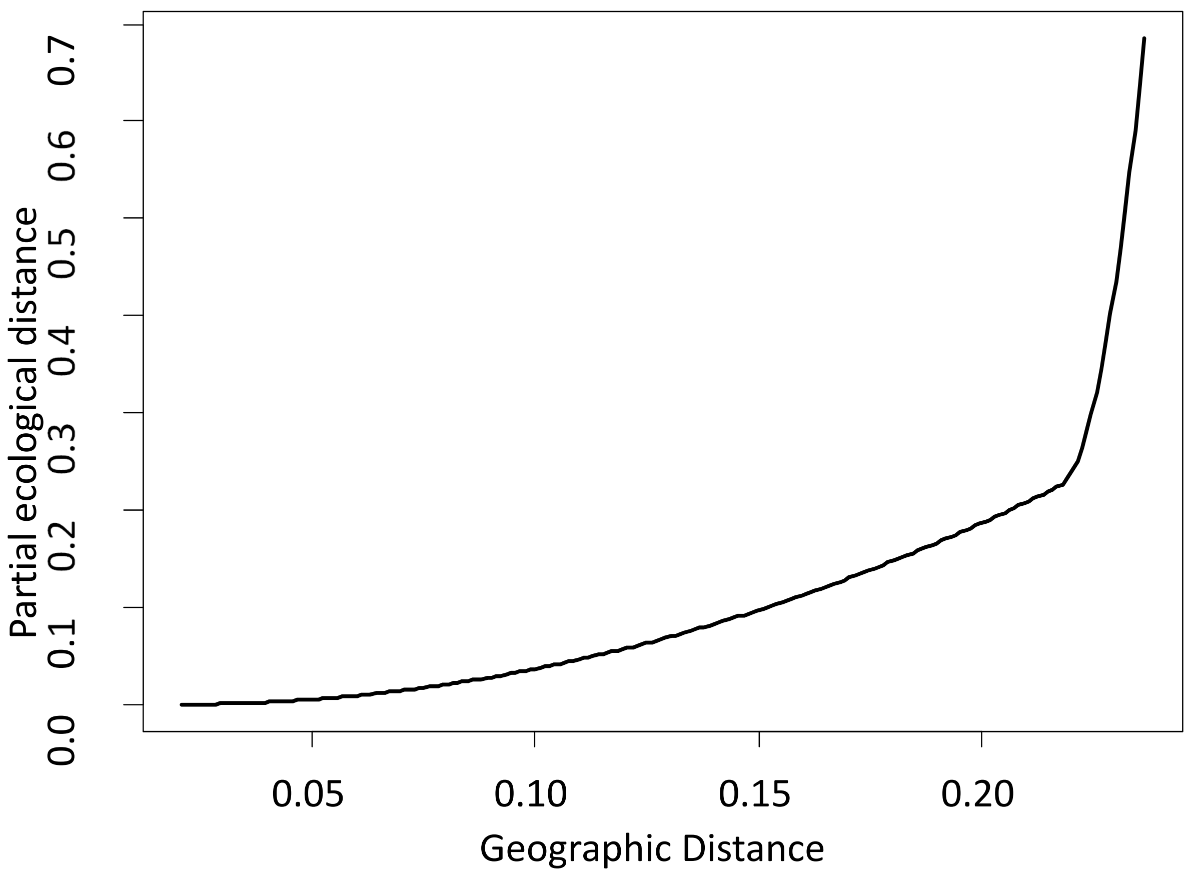

Figure S1. Generalized dissimilarity model-fitted I-spline (partial regression fit) of geographical distance and ecological distance.
